# Microbiome differences between wild and aquarium whitespotted eagle rays (*Aetobatus narinari*)

**DOI:** 10.1186/s42523-022-00187-8

**Published:** 2022-05-23

**Authors:** Ana G. Clavere-Graciette, Mary E. McWhirt, Lisa A. Hoopes, Kim Bassos-Hull, Krystan A. Wilkinson, Frank J. Stewart, Zoe A. Pratte

**Affiliations:** 1 School of Biological Sciences, Georgia Institute of Technology, Atlanta, GA USA; 2 Department of Microbiology & Cell Biology, Montana State University, Bozeman, MT USA; 3 Department of Research and Conservation, Georgia Aquarium, Atlanta, GA USA; 4 Sharks and Rays Conservation Research Program, Mote Marine Laboratory, Sarasota, FL USA; 5 Chicago Zoological Society’s Sarasota Dolphin Research Program, c/o Mote Marine Laboratory, Sarasota, FL USA

**Keywords:** Microbial community, Host-associated, Elasmobranch, Aquarium, Host health, Fish

## Abstract

**Background:**

Animal-associated microbiomes can be influenced by both host and environmental factors. Comparing wild animals to those in zoos or aquariums can help disentangle the effects of host versus environmental factors, while also testing whether managed conditions foster a ‘natural’ host microbiome. Focusing on an endangered elasmobranch species—the whitespotted eagle ray *Aetobatus narinari*—we compared the skin, gill, and cloaca microbiomes of wild individuals to those at Georgia Aquarium. Whitespotted eagle ray microbiomes from Georgia Aquarium were also compared to those of cownose rays (*Rhinoptera bonasus*) in the same exhibit, allowing us to explore the effect of host identity on the ray microbiome.

**Results:**

Long-term veterinary monitoring indicated that the rays in managed care did not have a history of disease and maintained health parameters consistent with those of wild individuals, with one exception. Aquarium whitespotted eagle rays were regularly treated to control parasite loads, but the effects on animal health were subclinical. Microbiome α- and β-diversity differed between wild versus aquarium whitespotted eagle rays at all body sites, with α-diversity significantly higher in wild individuals. β-diversity differences in wild versus aquarium whitespotted eagle rays were greater for skin and gill microbiomes compared to those of the cloaca. At each body site, we also detected microbial taxa shared between wild and aquarium eagle rays. Additionally, the cloaca, skin, and gill microbiomes of aquarium eagle rays differed from those of cownose rays in the same exhibit. Potentially pathogenic bacteria were at low abundance in all wild and aquarium rays.

**Conclusion:**

For whitespotted eagle rays, managed care was associated with a microbiome differing significantly from that of wild individuals. These differences were not absolute, as the microbiome of aquarium rays shared members with that of wild counterparts and was distinct from that of a cohabitating ray species. Eagle rays under managed care appear healthy, suggesting that their microbiomes are not associated with compromised host health. However, the ray microbiome is dynamic, differing with both environmental factors and host identity. Monitoring of aquarium ray microbiomes over time may identify taxonomic patterns that co-vary with host health.

**Supplementary Information:**

The online version contains supplementary material available at 10.1186/s42523-022-00187-8.

## Background

It is now widely accepted that host-associated microbiomes play critical roles in controlling host metabolism, physiology, behavior, and overall health [1–4]. Both environmental and host factors affect microbiome assembly [5] and vary in their relative influence based on factors such as host species and body site niche (e.g., skin, gills, or cloaca) [6–8]. In studying the effects of host species and environment on microbiome assembly, individuals under managed care, such as in zoos and aquariums, can be particularly useful given that these settings typically have tightly controlled environmental conditions. Moreover, these environments are relatively simple, with fewer variables compared to the wild, and the health of animals under managed care is often rigorously monitored. Species comparisons can also prove useful, as different species within the same space are exposed to the same conditions, removing environmental variables not possible in the wild. Therefore, animals under managed care provide an ideal framework for identifying microbial players that may be strongly related to host metabolism and health, as well as identifying potentially important drivers influencing microbiome assembly such as environmental change. Specifically, determining what characterizes a healthy versus dysbiotic (unhealthy or disrupted) state may help identify and address disease progression [9], leading to information that can enhance protection plans in the wild and veterinary care plans for zoos and aquariums [10, 11].

In marine systems, large predatory fish are sentinels of ecosystem health and are increasingly targeted as subjects for microbiome research due to their popularity in aquariums, longer lifespans, key roles in food webs, propensity for accumulating toxins in tissues and fat, and potential for carrying pathogenic microbes that can affect both human and environmental health [12–16]. In particular, elasmobranchs (sharks, rays and skates) are keystone species in many ecosystems in which they fill several roles in food chains as top- and meso-predators [17, 18], and declines in elasmobranch populations can have dramatic effects on ecosystem function [19]. While large sharks have received increased attention due to their prominent role as top predators [20–23], mesopredators, such as rays, are far less studied.

Whitespotted eagle rays (*Aetobatus narinari*) are mesopredatory batoids that play a key role in tropical and warm-temperate coastal water food webs, filling an intermediate position where this species acts as both predator (consuming gastropods and other benthic mollusks) [24, 25] and prey (for sharks and marine mammals) [26, 27]. Whitespotted eagle ray populations have largely declined due to overfishing and bycatch [28–33], and the species is now considered endangered by the International Union for Conservation of Nature. Despite the trophic importance of whitespotted eagle rays and the current decline of ray populations, studies on these animals remain scarce.

Whitespotted eagle rays can be kept and studied in zoos and aquaria. At Georgia Aquarium (Atlanta), whitespotted eagle rays have been housed for over a decade in a large exhibit alongside other ray species, whale sharks, and thousands of smaller fishes. The eagle rays are charismatic and popular with aquarium guests, helping raise awareness about elasmobranchs and their environments. Georgia Aquarium also collaborates with capture-tag-release studies of wild spotted eagle rays. Access to whitespotted eagle rays under managed care at Georgia Aquarium, in combination with collections of samples from wild populations, provides a unique opportunity to compare microbial structure in aquarium versus wild individuals. This comparison can help pinpoint potentially important prokaryotes conserved between aquarium and wild populations, particularly at external (skin and gill) and internal (cloaca/gut) body sites that have been shown in other fishes to harbor unique microbiomes tied to host health [6, 7] Additionally, comparing the microbiomes of different ray species in the same exhibit can reveal how host identity influences microbiome composition. In this study, we compared the taxonomic composition of gill, skin, and cloaca microbiomes of wild whitespotted eagle rays sampled near Sarasota, Florida to that of individuals under managed care at Georgia Aquarium. Whitespotted eagle ray microbial communities were also compared to those from aquarium cownose rays (*Rhinoptera bonasus*) to test the effect of host identity. We hypothesized that ray microbiomes would differ between aquarium and wild individuals, demonstrating an environmental influence on the ray microbiome, and between ray species in the same exhibit, demonstrating a host phylogenetic influence on the ray microbiome, with these differences varying in magnitude according to body site.

## Materials and methods

### Sample collection

**Fig. 1:**
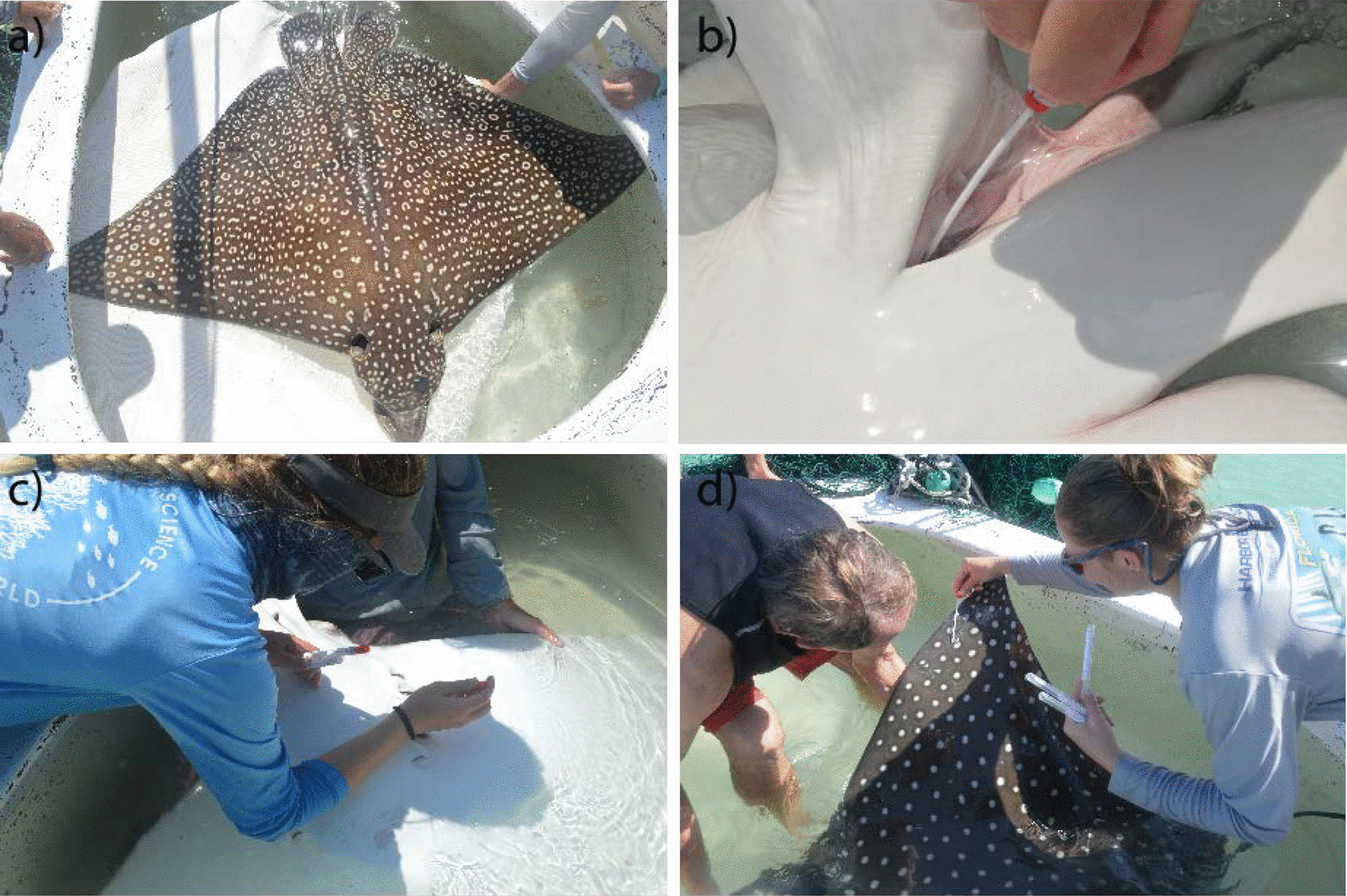
Pictures demonstrating sample collection for wild whitespotted eagle rays (*Aetobatus narinari*): **a** a whitespotted eagle ray in holding tank after being brought onboard the boat, **b** cloaca sampling, **c** gill sampling, **d** skin sampling. All samples were collected by gently rubbing sterile swabs along the target body site

Microbiome swabs were collected (described below) from a total of 18 wild whitespotted eagle rays in collaboration with Mote Marine Laboratory in Sarasota, Florida, and 15 aquarium-housed whitespotted eagle rays and 7 cownose rays in collaboration with Georgia Aquarium (Fig. 1). Location (wild vs. aquarium), date of sampling, sex, disc width, and weight for all individuals are provided in Table 1 and Additional file 1: Table S1. Wild individuals were captured, sampled, and released between June 2018 and April 2019 following protocols outlined in Bassos-Hull et al. [34] in Sarasota Bay (27.4 N, 82.6 W). Whitespotted eagle rays and cownose rays under managed care were sampled from the Ocean Voyager (OV) exhibit at Georgia Aquarium between 2018 and 2019. All aquarium rays were long-term residents of Georgia Aquarium, acquired between 2009 and 2018.

**Table 1:** Summary of the number of samples collected and the number of individuals sampled according to body site, ray species, and location

Sample type	Species	Location	Number of samples	Number of individuals represented
Cloaca	*Aetobatus narinari*	Aquarium	16	14
Gill	*Aetobatus narinari*	Aquarium	15	13
Skin	*Aetobatus narinari*	Aquarium	17	13
Cloaca	*Aetobatus narinari*	Wild	11	11
Gill	*Aetobatus narinari*	Wild	19	19
Skin	*Aetobatus narinari*	Wild	19	19
Cloaca	*Rhinoptera bonasus*	Aquarium	7	7
Gill	*Rhinoptera bonasus*	Aquarium	7	7
Skin	*Rhinoptera bonasus*	Aquarium	7	7
Water	NA	Aquarium	9	NA
Water	NA	Wild	3	NA

Georgia Aquarium’s OV exhibit is a 6.3 million gallon artificial seawater (Atlanta tap water mixed with Instant Ocean, Blacksburg, VA, USA) tank containing thousands of fish representing over 50 species from the open ocean. The water from the exhibit is filtered through a system composed of foam fractionators (protein skimmers), sand filters, ozone contact towers, countercurrent heat exchangers, sulfur-based denitrification vessels, and a deaeration tower, at a rate of 130,000 gallons per min, turning over approximately 4 million gallons once per hour [35]. Physico-chemical parameters of the exhibit are kept within a tight range with a temperature of 24 °C and salinity of 33 ppt. Approximately 225 kg of food goes into OV daily, 75 kg of which is broadcast into the system, while the remaining 150 kg is target fed to specific individuals such as the eagle rays. Eagle rays are target fed a daily ration of 1.3–1.5% their body weight consisting of surf clam (*Spisula solidissima*), hard-shell clam (*Mercenaria mercenaria*), Jonah crab (*Cancer borealis*), knobbed whelk (*Busycon carica*), Atlantic sea scallops (*Placopecten magellanicus*), whiteleg shrimp (*Litopenaeus vannamei*) and blue mussels (*Mytilus edulis*). In contrast, cownose rays are broadcast fed capelin (*Mallotus villosus*), Atlantic herring (*Clupea harengus*), mackerel (*Scomber scombrus*), surf clam, whiteleg shrimp, and night smelt (*Sprinchus starksi*), which is pumped to the bottom of the exhibit once or twice a day.

Samples were collected from aquarium eagle rays and cownose rays during routine veterinary examinations performed at least once a year or more frequently as veterinary or husbandry needs dictated. For these examinations, animals were transferred from the OV exhibit into a small holding pool with oxygenated water. Veterinary examinations included a visual health assessment and blood sampling for complete white blood cell counts and differentials, and a biochemistry panel. All parameters were within acceptable healthy ranges for all animals for the duration of this study. For microbiome sample collection, the rays were lightly restrained for the dorsal skin swab sampling and then rolled into dorsal recumbency to induce tonic immobility for gill and cloacal swab collection. Swabs were collected by gently rubbing sterile swabs along the gill, skin, or inside the cloaca, collecting mucus and microbes over the entire surface of the swab. Cloaca swabs were collected by placing the swab at least two inches into the cloaca and swirling the swab at the base of the spiral colon. Swabs were placed into 2 mL cryovials containing 1 mL of RNA/DNA stabilizing solution (25 mM sodium citrate, 10 mM EDTA and 70 g ammonium sulfate per 100 ml solution, pH 5.2) and stored at − 80 °C until lab processing. After examination, the rays were released into the OV exhibit and often resumed feeding the same day. Because microbiome samples were collected during routine veterinary examinations, and the frequency of these exams varied between individuals, certain individuals were sampled more than once during the same sampling period. For wild whitespotted eagle ray sample collection, rays were circled with a seine net, brought onboard the boat, and held in a water-filled, oxygenated tank while samples were collected in a manner similar to that described for Georgia Aquarium individuals.

Water samples were obtained from both the OV and wild environment to explore how seawater microbiome composition influences that of microbiomes on the ray body. Water samples from the OV water column were collected by filtration through 0.2 μm Sterivex filters and placed in RNA/DNA stabilizing solution (as described in [35]) as part of a 3-year time series monitoring program involving biweekly collections; the water column samples analyzed here correspond to those collected nearest in time (within 2 weeks) to each animal sampling event. Water samples from the wild environment were collected in 2018 at 3 locations in the same area where the animals were sampled. These water samples were filtered through a 0.2 μm Isopore membrane filter (Millipore) at the collection site, with each filter then placed in a 2 mL cryovial containing 1 mL of RNA/DNA stabilizing solution and stored at − 80 °C until lab processing, as in [36].

DNA was extracted from each swab or Isopore filter (water samples) by transferring the swabs/filters directly into Powerbead tubes from the Qiagen DNeasy PowerSoil DNA extraction kit following the manufacturer’s instructions. Extraction blanks (no swab or material added) were performed for each new kit. DNA was extracted from Sterivex filters using a phenol–chloroform method described in [35]. For each sample, the V4 region of the 16S rRNA gene was amplified by polymerase chain reaction (PCR) using the primers F515 and R806 [37], each appended with barcodes and Illumina-specific adapters as described previously [38]. Reaction mixtures included 2–5 μL DNA template, 12.5 μL Hot Start Taq PCR MasterMix (VWR), 0.25 μL (each) forward and reverse primers (20 μm), and 0.5 μl bovine serum albumin (20 mg/ml; New England BioLabs Inc.). PCR conditions included an initial 1 min denaturation at 94 °C, followed by 30 cycles of denaturation at 94 °C (1 min), primer annealing at 55 °C (2 min), and primer extension at 72 °C (90 s) and then a final extension at 72 °C for 10 min. The amplicon products were pooled at equimolar concentrations and purified with Diffinity RapidTip2 PCR purification tips (Diffinity Genomics, NY). Amplicons were sequenced on an Illumina MiSeq machine across 4 different runs, using a V2 500-cycle kit (250 × 250 bp) with 5% PhiX to increase read diversity.

### Illumina data processing

Sequence data were analyzed using DADA2 [39] and QIIME 2 2019.4 [40]. Raw sequences were demultiplexed, quality filtered, trimmed to 150 bp, denoised, and checked for chimeras following the DADA2 pipeline from [39]. Taxonomy was assigned to amplicon sequence variants (ASVs) using the SILVA-132 database. The resulting representative sequences, taxonomy and ASV tables, were imported into QIIME 2 2019.4 [40]. All ASVs were aligned with Mafft [41], via q2‐alignment, and used to construct a phylogeny with fasttree2 [42], via q2‐phylogeny. All raw data are publicly available at NCBI's SRA database under BioProject PRJNA712488.

### Quality control

Sequences classified as Chloroplast or Mitochondria were removed from all samples. Extraction blanks were processed following the procedures described above. One of these blanks was dominated by *Mollicutes*. As a result, *Mollicutes*-affiliated amplicons were removed from all samples, and the contaminated kit was not used again. All other extraction blanks did not pass quality control, validating our quality control method (described below).

Surface swabs of marine animals tend to produce low DNA yields, which can result in higher stochasticity in taxonomic composition among replicate samples. To address this, our sample set contained biological replicates (two separate samples taken from the same individual and body site at the same sampling event) and technical replicates (same sample, either amplified twice, or sequenced twice). To identify instances of high-replicate stochasticity, weighted UniFrac [43] distances among replicates (biological and technical) from the same sample were calculated using the q2‐diversity plugin using a rarefaction depth of 1150 reads. Weighted UniFrac distances among replicates were plotted in a boxplot. Samples representing points above Q3 were considered outliers and removed from the analysis; this resulted in the removal of one sample with four replicates, as well as the extraction blanks. After removing these outliers, replicates were merged for each sample to increase rarefaction depth to 1500. All subsequent analyzes were performed using this merged ASV table.

### Statistical analysis

After quality control and merging replicates, 139 samples remained. A summary of the samples in the final dataset and the associated metadata can be found in Additional file 1: Tables S1 and S2. For those rays that were sampled multiple times, sampling events were a minimum of four months apart (Additional file 1: Table S1). Given the wide span of time between the sampling events, we treated each sample as independent for all statistical analyses. To verify the independence of each sample taken from the same individual over time, we utilized Bray–Curtis dissimilarity matrices to calculate the average dispersion among samples from individuals repeatedly sampled and statistically compared the average dispersion to those individuals that were not repeatedly sampled. The average dispersion among those samples from individuals repeatedly sampled was similar to, and not significantly different from (p > 0.05), the average dispersion of those individuals not repeatedly sampled, indicating that samples from the same individuals can be treated as independent. α ‐diversity metrics (observed ASVs, Shannon Diversity Index, Pielou's Evenness, and Faith’s Phylogenetic Diversity Index [44]) and a Kruskal–Wallis test were computed using the q2-diversity plugin in QIIME2 to identify significant differences among aquarium whitespotted eagle rays, wild whitespotted eagle rays, and aquarium cownose rays, for each body site. β-diversity dissimilarity matrices (Bray–Curtis, weighted and unweighted UniFrac distances) were calculated using q2‐diversity plugin in QIIME2 and used to construct Principle Coordinate Analysis (PCoA) using Primer-e v.7 [45]. PERMANOVA and PERMDISP tests were subsequently performed to identify significant differences in microbiome composition and the level of inter-individual variability in microbiome composition among aquarium whitespotted eagle rays, wild whitespotted eagle rays, and aquarium cownose rays, for each body site. α ‐and β-diversity metrics were also evaluated for significant differences in the date of sampling, weight category, disc width category, and sex.

To identify the number of ASVs shared between different samples, Venn diagrams were constructed in Python using the package matplotlib-venn 0.11.5 with the merged, rarefied ASV table. Venn diagram calculations were performed to assess the number of ASVs shared with seawater. ASVs shared between seawater and host microbiomes were removed to assess the number of ASVs shared between body sites, animals from different environments (wild vs. aquarium), and species (whitespotted eagle ray vs. cownose ray). For each body site, we identified ASVs that differ in abundance between aquarium and wild individuals using the package DESeq2 in R [46] with the non-rarefied merged ASV table. For this analysis, we removed ASVs that were detected in both the host-associated and seawater microbiomes. Shared ASVs between aquarium and wild whitespotted eagle rays were determined as ASVs present in at least one individual from the aquarium and wild groups but not shared with the microbiome of the surrounding water.

## Results

Whitespotted eagle ray and cownose ray behavior and feeding were closely monitored at Georgia Aquarium, and animals were handled routinely for veterinary examination. All rays appeared healthy and had normal blood panels throughout the sampling period, with the exception of one whitespotted eagle ray (Additional file 1: Table S1). During this period, aquarium eagle rays were treated for monocotylid monogenes present on the gills of the animals; impacts to animal health were subclinical (no detectable signs of disease), and monogene parasites are also common in wild eagle rays [46–48], although parasite loads in wild individuals have yet to be quantified in the scientific literature. Likewise, all wild whitespotted eagle rays captured in Sarasota Bay were examined and showed no obvious signs of disease.

After quality filtering, trimming, merging of replicates, and rarefaction, 139 samples remained in the final dataset, representing 18 wild whitespotted eagle rays, 15 aquarium-housed whitespotted eagle rays, 7 cownose rays, 3 wild water samples, and 9 OV water samples (Table 1; Additional file 1: Table S1). From these, we detected a total of 5398 amplicon sequence variants (ASVs). 1916 ASVs were detected in aquarium whitespotted eagle rays (cloaca—549; gills—1188; skin—1118); 1694 ASVs were detected in aquarium cownose rays (cloaca—738; gills—1006; skin 803); and 3031 ASVs were detected in wild whitespotted eagle rays (cloaca—909; gills—1785; skin—2143). These ASV numbers include some ASVs shared between body sites, species, and locations (aquarium vs. wild). Rarefaction curves are in Additional file 11: Fig. S1.

### Aquarium versus wild whitespotted eagle rays

**Fig. 2:**
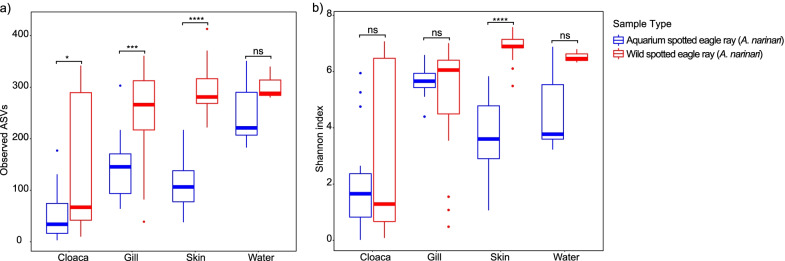
α-Diversity metrics for **a** observed amplicon sequence variants (ASVs), and **b** Shannon diversity indices, for different body sites (cloaca, gill, and skin) of aquarium and wild whitespotted eagle rays (*Aetobatus narinari*), along with their respective water samples. Aquarium whitespotted eagle rays have lower diversity for all body sites compared to wild whitespotted eagle rays

**Fig. 3:**
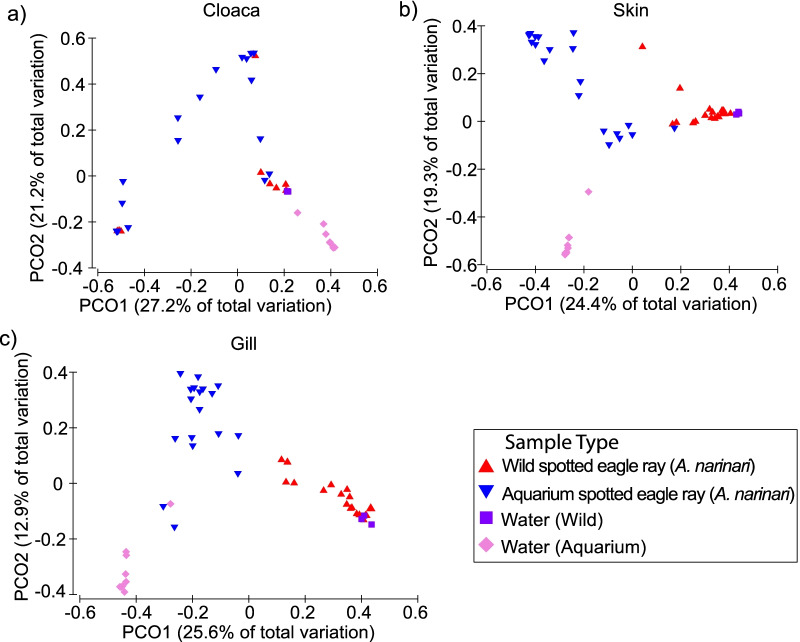
Principal coordinate analysis (PCoA) of β-diversity comparison using Bray–Curtis distances between wild whitespotted eagle rays (*Aetobatus narinari*), and aquarium whitespotted eagle rays (*Aetobatus narinari*) for **a** cloaca, **b** gill, and **c** skin samples. Note that wild whitespotted eagle rays harbor different microbial communities than aquarium whitespotted eagle rays, and both aquarium and wild whitespotted eagle rays harbor microbial communities that differ from the surrounding water for all body sites. Interestingly, cloaca samples show higher overlap, suggesting higher similarities compared to more external body sites (gill and skin)

For each body site, microbiomes of aquarium eagle rays had significantly fewer observed ASVs compared to those of wild individuals (Fig. 2; Table 2). The same general pattern was observed using various α-diversity indexes, although not all comparisons involved statistically significant differences (Table 2). Similarly, for each body site, whitespotted eagle ray microbiome composition (β-diversity) differed significantly between aquarium and wild individuals (Fig. 3; Table 3), except for the cloaca microbial composition, which was more similar between aquarium and wild individuals compared to other body sites (Fig. 3; Additional file 1: Fig. S2; Table 3). Dispersion analyses measuring the level of inter-individual variability showed no significant differences in cloaca microbial community composition between aquarium and wild whitespotted eagle rays, while some dispersion analyses between aquarium and wild gill and skin microbiomes involved significant differences depending on β-diversity metric (Table 4). For all aquarium and wild whitespotted eagle rays, no significant compositional differences were associated with date of sampling, weight category, disc width category, or sex (data not shown).

**Table 2:** Pairwise results for a one-way analysis of variance (Kruskal–Wallis) for all α-diversity metrics between aquarium cownose (*Rhinoptera bonasus*) and aquarium and wild whitespotted eagle rays (*Aetobatus narinari*) for different body sites

Comparison	Diversity index	Cloaca	Gill	Skin	Water
Aquarium whitespotted eagle ray (C) × wild whitespotted eagle ray (W)	Observed	* W > C	*** W > C	**** W > C	NS
	Shannon	NS	NS	**** W > C	NS
	Evenness	NS	* C > W	*** W > C	NS
	Faith’s	* W > C	** W > C	**** W > C	NS
Aquarium whitespotted eagle ray (C) × aquarium cownose ray (Co)	Observed	* Co > C	** Co > C	* Co > C	NA
	Shannon	* Co > C	* Co > C	*** Co > C	NA
	Evenness	* Co > C	NS	** Co > C	NA
	Faith’s	* Co > C	* Co > C	* Co > C	NA

*A significant difference p ≤ 0.05; **p ≤ 0.01; ***p ≤ 0.001; ****p ≤ 0.0001, and NS indicates not significant (p > 0.05). NA indicates no possible comparison. α-diversity metrics include observed amplicon sequence variants (ASVs), Shannon diversity index, Pielou’s evenness, and Faith’s Phylogenetic Diversity

**Table 3:** Pairwise results for all permutational multivariate analysis of variance (PERMANOVA) for all β-diversity metrics between aquarium cownose (*Rhinoptera bonasus*) and aquarium and wild whitespotted eagle rays (*Aetobatus narinari*) for different body sites

Comparison	Diversity index	Cloaca	Gill	Skin	Water
Aquarium whitespotted eagle ray × wild whitespotted eagle ray	Bray–Curtis	NS	p ≤ 0.01	p ≤ 0.001	p ≤ 0.001
	Weighted	NS	p ≤ 0.01	p ≤ 0.01	p ≤ 0.001
	Unweighted	p ≤ 0.01	p ≤ 0.001	p ≤ 0.001	p ≤ 0.001
Aquarium whitespotted eagle ray × aquarium cownose ray	Bray–Curtis	NS	p ≤ 0.01	p ≤ 0.001	NA
	Weighted	p ≤ 0.05	p ≤ 0.05	p ≤ 0.01	NA
	Unweighted	p ≤ 0.01	p ≤ 0.001	p ≤ 0.001	NA

NS indicates not significant (p > 0.05). NA indicates no possible comparison. β-diversity metrics include Bray–Curtis, weighted UniFrac, and unweighted UniFrac

**Table 4:** Pairwise results for all permutational multivariate analysis of dispersion (PERMDISP) for all β-diversity metrics and body sites for wild and aquarium whitespotted eagle rays (*Aetobatus narinari*) and aquarium cownose rays (*Rhinoptera bonasus*)

Comparison	Diversity index	Cloaca	Gill	Skin	Water
Aquarium whitespotted eagle ray × wild white-spotted eagle ray	Bray–Curtis	NS	p ≤ 0.05	NS	NS
	Weighted	NS	NS	p ≤ 0.01	NS
	Unweighted	NS	NS	p ≤ 0.05	p ≤ 0.05
Aquarium whitespotted eagle ray × aquarium cownose ray	Bray–Curtis	NS	NS	NS	NA
	Weighted	NS	NS	NS	NA
	Unweighted	NS	NS	NS	NA

NS indicates not significant (p > 0.05). NA indicates no possible comparison. β-diversity metrics include Bray–Curtis, Weighted UniFrac, and Unweighted UniFrac

Across both aquarium and wild individuals, the most abundant bacteria in the cloaca included a single ASV classified as *Photobacterium damselae* and several ASVs from the Order *Flavobacteriales*, constituting 23% (± 31) and 34% (± 43) of total sequences, respectively (Additional file 1: Fig. S3). Differential abundance analyses performed using DESeq2 showed that most microbial ASVs that differed significantly in frequency between wild and aquarium whitespotted eagle rays were also found in the surrounding water (Additional file 1: Fig. S4). These included *SAR11*-clade Ia, *Synechococcus*-CC9902, and *Tyzzerella sp.*, which were more abundant in wild whitespotted eagle rays, and *Helcococcus sp.*, which was more abundant in aquarium rays (Additional file 1: Fig. S3). After removing those ASVs shared with the water column, no difference in abundance was found in cloaca ASVs between wild and aquarium whitespotted eagle rays. Only one ASV, identified as *Kistimonas sp.,* was significantly enriched in the gills of wild versus aquarium whitespotted eagle rays. This ASV was present in two wild individuals (at 12.5% and 34.3%) and absent from the gills of aquarium individuals. Only one ASV, identified as *Alkanindiges illinoisensis,* was significantly enriched in the skin of aquarium versus wild whitespotted eagle rays. This ASV was present in half of aquarium individuals, but at an average relative abundance of only 0.3%, and was absent in all wild individuals.

### Aquarium whitespotted eagle versus cownose ray

Prokaryotic richness and β-diversity of aquarium cownose rays were higher compared to those of aquarium whitespotted eagle rays for all body sites (Additional file 1: Fig. S5). Microbiomes of aquarium cownose rays clustered apart from those of aquarium and wild whitespotted eagle rays, although of the two, cownose rays were more similar to those of aquarium whitespotted eagle rays (Additional file 1: Fig. S2; Table 3). Dispersion (i.e., inter-sample variation) did not differ between aquarium cownose rays and aquarium whitespotted eagle rays for all body sites (Table 4).

When evaluated at the phylum level, microbial composition did not differ between cownose rays and whitespotted eagle rays housed in the same exhibit, being dominated in both ray species by *Proteobacteria*, *Bacteroidetes*, and *Firmicutes*. *Bacteroidetes* was abundant in the cloaca, while *Cyanobacteria* and *Actinobacteria* had higher proportions in the gills and skin where *Proteobacteria* was also abundant. At finer taxonomic levels, the cownose ray microbiome was distinct from that of the whitespotted eagle ray. Significant differences were observed in the cloaca and skin microbiomes where unclassified species from the genus *Kordiimonas* and the class *Rhodobacteraceae* were at least two times more abundant in aquarium cownose rays compared to aquarium whitespotted eagle rays (Additional file 1: Fig. S3).

### Differences in body site niches

**Fig. 4:**
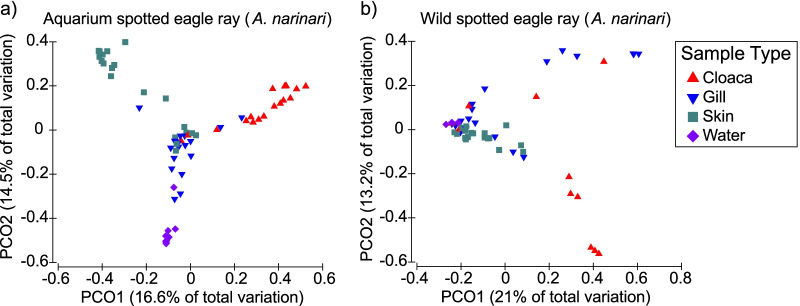
Principal coordinate analysis (PCoA) based upon Bray–Curtis dissimilarity matrices between cloaca, gill, skin, and water for **a** aquarium whitespotted eagle rays (*Aetobatus narinari*), and **b** wild whitespotted eagle rays. Note that different body sites have different microbial community structures that differ from the surrounding water, as indicated by the separate clustering. In aquarium whitespotted eagle rays, the gills appear more similar to the surrounding water, while the skin is more similar to the surrounding water in wild whitespotted eagle rays

Using datasets partitioned by ray species and location (aquarium whitespotted eagle rays, wild whitespotted eagle rays, and aquarium cownose rays), we analyzed microbiome composition among body sites. For each ray group, cloaca microbiomes were significantly less diverse in terms of α-diversity compared to those of other body sites (Additional file 1: Fig. S5; Table 5) and water (Table 6). The microbial composition of whitespotted eagle ray microbiomes also varied among body sites (Fig. 4; Table 6); this was not true for cownose ray microbiomes (Additional file 1: Fig. S6; Table 6). In PCoA plots, the microbiomes of the gills and skin for both whitespotted eagle and cownose rays were more similar to those of the surrounding environment than those of wild whitespotted eagle rays (Fig. 4; Additional file 1: Figs. S2 and S6), but still remained significantly distinct from each other (Table 6). However, the microbiome of wild whitespotted eagle rays was not always statistically distinct from that of seawater, depending on the metric used (Table 6).

**Table 5:** Pairwise results for a one-way analysis of variance (Kruskal–Wallis) for all α-diversity metrics between different body sites for aquarium cownose (*Rhinoptera bonasus*) and wild and aquarium whitespotted eagle rays (*Aetobatus narinari*)

Comparison	Diversity index	Aquarium whitespotted eagle ray	Wild whitespotted eagle ray	Aquarium cownose ray
Cloaca (C) × gill (G)	Observed	*** G > C	NS	* G > C
	Shannon	**** G > C	NS	* G > C
	Evenness	**** G > C	NS	NS
	Faith’s	*** G > C	NS	NS
Cloaca (C) × skin (S)	Observed	** S > C	* S > C	NS
	Shannon	** S > C	** S > C	NS
	Evenness	* S > C	** S > C	* S > C
	Faith’s	** S > C	NS	NS
Gill (G) × skin (S)	Observed	NS	NS	* G > S
	Shannon	**** G > S	*** S > G	NS
	Evenness	*** G > S	**** S > G	NS
	Faith’s	NS	NS	NS

*A significant difference p ≤ 0.05; **p ≤ 0.01; ***p ≤ 0.001; ****p ≤ 0.0001, and NS indicates not significant (p > 0.05). NA indicates no possible comparison. α-diversity metrics include observed amplicon sequence variants (ASVs), Shannon diversity index, Pielou’s evenness, and Faith’s Phylogenetic Diversity

**Table 6:** Pairwise results for all permutational multivariate analysis of variance (PERMANOVA) for all β-diversity metrics between different body sites for cownose (*Rhinoptera bonasus*) and wild and aquarium whitespotted eagle rays (*Aetobatus narinari*)

Comparison	Diversity index	Aquarium whitespotted eagle ray	Wild whitespotted eagle ray	Aquarium cownose ray
Cloaca × gill	Bray–Curtis	p ≤ 0.001	p ≤ 0.01	NS
	Weighted	p ≤ 0.001	p ≤ 0.01	NS
	Unweighted	p ≤ 0.001	p ≤ 0.01	NS
Cloaca × skin	Bray–Curtis	p ≤ 0.001	p ≤ 0.01	NS
	Weighted	p ≤ 0.001	p ≤ 0.01	NS
	Unweighted	p ≤ 0.001	p ≤ 0.01	NS
Gill × skin	Bray–Curtis	p ≤ 0.001	p ≤ 0.01	NS
	Weighted	p ≤ 0.001	p ≤ 0.01	NS
	Unweighted	p ≤ 0.001	p ≤ 0.01	NS
Cloaca × water	Bray–Curtis	p ≤ 0.001	NS	p ≤ 0.01
	Weighted	p ≤ 0.001	NS	p ≤ 0.01
	Unweighted	p ≤ 0.001	NS	p ≤ 0.01
Gill × water	Bray–Curtis	p ≤ 0.001	NS	p ≤ 0.01
	Weighted	p ≤ 0.001	p ≤ 0.05	p ≤ 0.01
	Unweighted	p ≤ 0.001	p ≤ 0.05	p ≤ 0.01
Skin × water	Bray–Curtis	p ≤ 0.001	NS	p ≤ 0.01
	Weighted	p ≤ 0.001	NS	p ≤ 0.01
	Unweighted	p ≤ 0.001	p ≤ 0.01	p ≤ 0.01

NS indicates no significant (p > 0.05)

### Shared microbiome between aquarium and wild whitespotted eagle rays

We identified ASVs shared between aquarium and wild whitespotted eagle rays, defined as ASVs found in at least one ray from the aquarium and one ray from the wild, excluding ASVs that were also found in seawater. We found 72, 216, and 306 shared ASVs in the cloaca, gill, and skin microbiomes, respectively. While the number of cloaca ASVs shared between wild and aquarium whitespotted eagle rays was lower compared to shared gill and skin ASVs, shared cloaca ASVs represented a much larger proportion of the cloaca microbiome—3 to 11 times higher compared to gill or skin ASVs shared between aquarium and wild individuals (Additional file 1: Table S2). For wild whitespotted eagle rays, the fraction of ASVs shared between ray microbiomes and the surrounding seawater was similar across the three body sites (gill, skin, and cloaca) but substantially higher compared to the shared fraction calculated for the cloaca microbiome of aquarium whitespotted eagle rays (Additional file 1: Table S2). In aquarium cownose and whitespotted eagle rays, ASVs shared between the gills and skin were lower in numbers and relative abundances than those shared between the cloaca and gills or cloaca and skin (Additional file 1: Table S3).

The cloaca microbiome shared between wild and aquarium rays was primarily composed of *Proteobacteria*, *Bacteroidetes*, and *Firmicutes*. An ASV classified to the order *Flavobacteriales* represented an average of 39% and 26% of sequences in aquarium and wild cloacal datasets, respectively. Apart from this ASV, > 95% of the cloaca shared ASVs had frequencies under 2%. Similarly, the gill and skin shared microbiomes were primarily composed of *Proteobacteria*, *Firmicutes*, and *Bacteroidetes*, as well as *Actinobacteria*. An ASV classified to the order *Betaproteobacteriales* represented an average of 1% and 24% of sequences in aquarium and wild gill datasets, respectively. None of the shared ASVs of the gill represented over 3% of sequences in aquarium ray microbiomes. On the skin, an ASV identified as *Helcococcus sp.* represented an average of 32% and 2% of sequences in aquarium and wild datasets, respectively. A shared skin ASV classified as ‘unknown bacterium’ represented 8% of skin sequences from rays under managed care. All other shared skin ASVs had relative abundancies under 3%.

## Discussion

We characterized the microbiome of the whitespotted eagle ray, focusing on differences between aquarium and wild individuals. We found evidence that both environment and host identity influence microbial community composition, as discussed below.

### Environmental influence (aquarium vs. wild)

We found significantly lower α-diversity in aquarium whitespotted eagle ray microbiomes, compared to those of wild counterparts. Previous studies on marine and terrestrial animals also show decreases in α-diversity associated with human-managed environments [49–51]. In this case, the lower α-diversity under managed care may be driven by diversity found in the OV water column, which was low compared to that of seawater microbiomes surrounding the wild animals [35]. For example, bacteria common to the wild water samples, such as *Betaproteobacteriales*, *SAR11*, and *Synechococcus* were enriched in wild whitespotted eagle ray microbiomes at all body sites compared to microbiomes in aquarium animals, suggesting that many of these microbes are lost upon transfer of wild animals to aquarium systems. In animals under managed care at other zoos and aquaria, decreases in α-diversity are accompanied by microbiome compositional shifts (β-diversity) [49–52]. We observed such differences in β-diversity for both internal (cloaca) and external (skin and gills) body sites, although differences were less pronounced for the cloaca. These differences suggest that environment and possibly diet have varying degrees of influence on different body sites. While there is some concern that changes in microbiome diversity may cause shifts or losses in metabolic functions carried out by specific microbes [53], these changes are often host specific and hard to link to host metabolism and health. It may be that while microbiome composition differs between wild and aquarium individuals, the repertoire of microbial functional genes is similar. Metagenomic studies will help determine if functional differences exist between microbiomes of aquarium and wild individuals.

While the cloaca and fecal microbiome can be influenced by habitat changes [54, 55], this influence appears to be minimal in our study, with the cloaca microbiome niche being relatively stable compared to more external body sites. Microbiomes in all body niche sites differed significantly between aquarium and wild whitespotted eagle rays. However, in wild and aquarium animals, cloaca microbiomes were less diverse and more similar to one another than to those of other body sites; a similar pattern was also seen in teleost fish [56]. This conservation of microbiome structure is likely because the cloaca, despite its constant exposure to seawater microbiomes, also contains bacteria from feces. Fecal microbiomes may be relatively stable between wild and aquarium individuals, particularly given that the diet of aquarium animals is designed to be similar to that of wild rays. However, some significant differences were detected. Specifically, the proportional abundance of *Tyzzerella sp*. bacteria was over 300 times higher in the cloaca of aquarium versus wild whitespotted eagle rays. *Tyzzerella sp.* have been found in the gut microbiota of diverse animals [57–59] and appeared to vary with dietary shifts [57, 60]. In humans, higher proportions of *Tyzzerella* have been associated with low-fiber diets containing high levels of processed meat, fat, sugar, and sodium [61], although this association may not hold true for elasmobranchs.

Previous studies have shown that fish external surfaces such as the skin and gills can be strongly influenced by environmental processes [62, 63]. Among the body niches examined in our study, the skin microbiome was the most variable in composition between aquarium and wild eagle rays and was strongly influenced by the water microbiome. Sequences matching the genus *Helcococcus* were substantially enriched (> 10 ×) on whitespotted eagle rays in the aquarium, where this genus was also enriched in aquarium water compared to the natural seawater. After removing ASVs shared with seawater, the only ASV that differed significantly in abundance between the skin microbiome of aquarium and wild whitespotted eagle rays was classified as *Alkanindiges illinoisensis,* an alkane-degrading bacterium not previously reported in marine ecosystems [64–69]. Additionally, an uncultured prokaryote (sequence ID: MT067094.1) that shared 100% identity with our ASV and *Alkanindiges illnoisensis* was found in lake sediments [70]. Its role in the aquarium system and in ray microbiomes is unclear; however, it was present at low relative abundances (< 0.5%) in aquarium individuals. Only one ASV, identified as *Kistimonas,* was significantly more abundant in the gills of wild compared to aquarium rays. *Kistimonas* and *Kistimonas*-like species have been identified in marine invertebrate gill [71] and skin [72] microbiomes. While *Kistimonas* includes pathogenic species [73], we have no reason to believe that the ASV detected here is pathogenic.

### Species influence: whitespotted eagle ray versus cownose ray

Microbiome composition was broadly similar between co-habitating whitespotted eagle rays and cownose rays at all body sites, but also contained species-specific signatures, similar to that observed in teleost fishes [74]. Species-specific signatures were most pronounced in cloacal microbiomes. For example, an unidentified *Betaproteobacteriales* (incertae-sedis) ASV was present only in the cloaca of cownose rays, while *Tyzzerella sp.* was only present in whitespotted eagle rays. Such variation may be driven by diet or intestinal physiological differences between hosts, notably as these two ray species consume different, though nutritionally similar, prey items in the aquarium. In contrast, skin and gill microbiomes are relatively similar between ray species. After excluding ASVs shared with seawater, no ASVs with a relative abundance above 1% differed in representation between whitespotted eagle rays and cownose rays, raising the possibility that shared environmental conditions may drive convergence in external microbiomes [56, 75]. Interestingly, whitespotted eagle ray microbiomes differed in composition (β-diversity) among body sites, whereas those of cownose rays did not, suggesting that body site niche may have a smaller influence on microbiome composition in cownose rays. However, the cownose ray sample size was low (7 individuals), and additional studies may be needed to identify clear niche separation.

### Shared taxa

We defined shared ASVs as those ASVs detected in at least one wild and one aquarium individual. The number of ASVs shared between the cloaca of wild and aquarium whitespotted eagle rays was fewer compared to those shared between the cloaca and seawater. However, those few shared ASVs were dominant members of the community in both wild and aquarium individuals. This suggests that fewer ASVs with higher relative abundances compose the microbiome of the cloaca in whitespotted eagle rays, while less abundant environmental ASVs from the water column may be transient in the cloaca microbiome. For example, the shared cloaca microbe *Flavobacteriales* was the most abundant, contributing up to 99% of microbiome sequences in certain samples. *Flavobacteriales* have been recognized as part of the intestinal microbiome of trout [76] and other marine fish [77, 78]. Certain *Flavobacterium* species have also been associated with disease in fish [79, 80], although we have no evidence of disease in any of the individuals sampled here, suggesting these strains are not pathogenic. However, further studies are needed to determine the role of *Flavobacteriales* in the cloaca microbiome of whitespotted eagle rays.

*Photobacterium damselae* also dominated the cloaca microbial community of both wild and aquarium rays. It is important to point out that *Photobacterium* harbors multiple copies of the 16S rRNA gene [81], which may result in an overestimation of its representation in the microbiome. Despite the potential pathogenicity of this microbe [82], rays appeared healthy based on medical assessments, which suggests that the strain found in these animals may not be pathogenic. Indeed, this bacterium has been identified as a common constituent of healthy elasmobranch skin and blood microbiomes [83–85].

The Anna-Karenina principle may also be useful in estimating host health and/or dysbiosis. This principle suggests that microbiome perturbations translate into a variety of unstable states (i.e., high inter-sample dispersion) and not a single dysbiotic state [86]. We did not detect statistically significant variation in dispersion between cloaca microbiomes of wild versus aquarium animals, suggesting that managed care was not associated with microbiome dysbiosis. Moreover, known fish pathogens were not among the taxa significantly enriched in the cloacal microbiome of aquarium individuals compared to that of wild individuals, suggesting that aquarium whitespotted eagle rays are not less healthy than their wild counterparts.

Among the ASVs composing the shared gill microbiome, one ASV classified as *Betaproteobacteriales* was particularly abundant in wild whitespotted eagle rays. Members of the *Betaproteobacteriales* are ubiquitous in various aquatic environments [87, 88] and appear to be enriched in fish gill microbiomes [7]. Finally, as part of the skin microbiome shared between wild and aquarium whitespotted eagle rays, an ASV identified as *Helcococcus* was particularly enriched in aquarium individuals. This ASV shared 98% identity with an uncultured bacterium clone from California sea lion rectal swabs [89], as well as with an uncultured bacterium from the skin of fur seals [90]. In humans, *Helcococcus* has been associated with disease [91]. However, *Helcococcus* commonly colonizes diverse body sites in marine animals including the respiratory tract of dolphins [92, 93] and whales [94], and the gut of *Cephalopholis urodeta*, a carnivorous coral reef fish [95]. The contribution of *Helcococcus* to host health or physiology, if any, remains unknown.

## Conclusions

The microbiomes of whitespotted eagle rays under managed care at Georgia Aquarium had lower α-diversity and differed in community structure (β-diversity) compared to microbiomes of wild rays. However, the magnitude of these differences varied by body site niche. Compared to microbiomes of the gill and skin, the cloaca microbiome differed the least between aquarium and wild whitespotted eagle rays, suggesting that the diet of aquarium animals (clams, crab, whelk, scallops, shrimp, and mussels) is either similar to that of wild animals or that the effect of host physiology prevents a major change in cloaca microbiome structure. While wild individuals appeared healthy, their health status was not medically determined, nor was their age, and these factors may contribute to some of the variation we observe in this study. However, it is likely that all aquarium and wild animals sampled spanned diverse age groups, suggesting that the patterns observed are primarily driven by other parameters such as changes in environmental conditions. Finally, the microbiome of aquarium whitespotted eagle rays was different from that of cownose rays in the same exhibit, highlighting the influence of host related factors in ray microbial community assembly.

Our findings provide a framework for interpreting future data on ray microbiomes, helping identify signatures of a healthy microbiome as well as the extent to which such signatures vary due to environmental versus host factors. Future research should build upon this framework, for example by examining microbiome function and extending microbiome characterizations across gradients of host and ecosystem health. Such studies may identify microbial markers that can be used to guide managed care and conservation strategies.

## Supplementary Information

**Additional file 1:** Figures S1–S6 and Tables S1–S3 reporting sample identity and collection information, and additional microbiome diversity statistics.

## Data Availability

All raw data are publicly available at NCBI's SRA database under BioProject PRJNA712488.

## Abbreviations

OV: Ocean Voyager (exhibit at Georgia Aquarium)
DNA: Deoxyribonucleic acid
PCR: Polymerase chain reaction
ASV: Amplicon sequence variant
