# Supplementary material for: Microbiome differences between wild and aquarium whitespotted eagle rays (Aetobatus narinari)
Source: Anim Microbiome. 2022 May 23;4:34. doi: 10.1186/s42523-022-00187-8 (PMC9128078; doi:10.1186/s42523-022-00187-8)
Supplement: Supplementary file 1 — Additional file 1: Figures S1–S6 and Tables S1–S3 reporting sample identity and collection information, and additional microbiome diversity statistics. [file 42523_2022_187_MOESM1_ESM.docx]

Supplementary Tables and Figures for

**Microbiome differences between wild and aquarium whitespotted eagle rays (*Aetobatus narinari)***

Ana G. Clavere Graciette^a^, Mary E. McWhirt^a^, Lisa A. Hoopes^c^, Kim Bassos-Hull^d,e^, Krystan A. Wilkinson^d,e^, Frank J. Stewart^a,b^ , Zoe A. Pratte^a,b#^

**Affiliations**

^a^ School of Biological Sciences, Georgia Institute of Technology, Atlanta, GA, USA

^b^ Department of Microbiology and Immunology, Montana State University, Bozeman, MT, USA

^c^ Georgia Aquarium, Atlanta, GA, USA

^d^ Sharks and Rays Conservation Research Program, Mote Marine Laboratory, Sarasota, FL USA

^e^ Chicago Zoological Society’s Sarasota Dolphin Research Program, c/o Mote Marine Laboratory, Sarasota, FL USA

**Supplementary Tables**

**Supplementary Table 1.** Metadata for all samples, including aquarium and wild whitespotted eagle rays (*Aetobatus narinari*), and aquarium cownose rays (*Rhinoptera bonasus*). Not all measurements are known for all individuals. Veterinary treatment (praziquatel baths) for monocotylid monegenes present on the gills of aquarium eagle ray were common, but impacts to animal health were subclinical and these parasites are also common in wild eagle rays.

| Elasmobranch ID | Species | Location | Year arrived in exhibit | Date sample collected | Cloaca | Gill | Skin | Sex | Disc width (cm) | Weight (kg) | Notes |
| --- | --- | --- | --- | --- | --- | --- | --- | --- | --- | --- | --- |
| 585 | Whitespotted Eagle Ray | Wild | NA | 4/24/18 | Yes | Yes | Yes | F | 60-90 | 0-20 |  |
| 586 | Whitespotted Eagle Ray | Wild | NA | 4/24/18 | Yes | Yes | Yes | F | >150 | >60 |  |
| 584 | Whitespotted Eagle Ray | Wild | NA | 4/24/18 |  | Yes | Yes | M | 60-90 | 0-20 |  |
| 588 | Whitespotted Eagle Ray | Wild | NA | 4/26/18 |  | Yes | Yes | M | >150 | >60 |  |
| 587 | Whitespotted Eagle Ray | Wild | NA | 4/26/18 | Yes | Yes | Yes | M | >150 | >60 |  |
| 589 | Whitespotted Eagle Ray | Wild | NA | 4/27/18 | Yes | Yes | Yes | F | 90-120 | 20-40 |  |
| 590 | Whitespotted Eagle Ray | Wild | NA | 5/1/18 | Yes | Yes | Yes | F | 60-90 | 0-20 |  |
| 591 | Whitespotted Eagle Ray | Wild | NA | 5/3/18 | Yes | Yes | Yes | M | >150 | 40-60 |  |
| 592 | Whitespotted Eagle Ray | Wild | NA | 5/3/18 |  | Yes | Yes | F | 90-120 | 0-20 |  |
| 593 | Whitespotted Eagle Ray | Wild | NA | 5/3/18 |  | Yes | Yes | M | 120-150 | 20-40 |  |
| 594 | Whitespotted Eagle Ray | Wild | NA | 5/3/18 | Yes | Yes | Yes | M | 120-150 | 20-40 |  |
| 596 | Whitespotted Eagle Ray | Wild | NA | 5/4/18 |  | Yes | Yes | M | >150 | 40-60 |  |
| 595 | Whitespotted Eagle Ray | Wild | NA | 5/4/18 | Yes | Yes | Yes | M | >150 | 40-60 |  |
| 597 | Whitespotted Eagle Ray | Wild | NA | 5/9/18 |  | Yes | Yes |  |  |  |  |
| 603 | Whitespotted Eagle Ray | Wild | NA | 4/25/19 | Yes | Yes | Yes | F |  |  |  |
| 600 | Whitespotted Eagle Ray | Wild | NA | 4/23/19 |  | Yes | Yes | F |  |  |  |
| 604 | Whitespotted Eagle Ray | Wild | NA | 4/25/19 |  | Yes | Yes | F |  |  |  |
| 601 | Whitespotted Eagle Ray | Wild | NA | 4/23/19 | Yes | Yes | Yes | M |  |  |  |
| 605 | Whitespotted Eagle Ray | Wild | NA | 4/25/19 |  | Yes | Yes | M |  |  |  |
| E15030 | Whitespotted Eagle Ray | Aquarium | 2015 | 2/28/18  3/5/19 | Yes  Yes | Yes | Yes  Yes | F | 120-150 | 40-60 |  |
| E12070 | Whitespotted Eagle Ray | Aquarium | 2012 | 3/5/19 | Yes | Yes | Yes |  |  |  |  |
| E13090 | Whitespotted Eagle Ray | Aquarium | 2013 | 3/1/18  7/1/18 | Yes  Yes | Yes  Yes | Yes  Yes | M | 120-150 | 20-40 | Died soon after 7/1/18 sampling |
| E12071 | Whitespotted Eagle Ray | Aquarium | 2012 | 3/1/18  3/6/19 | Yes  Yes | Yes  Yes | Yes  Yes | M | 120-150 | 20-40 |  |
| E15029 | Whitespotted Eagle Ray | Aquarium | 2015 | 7/1/18  11/14/18  3/8/19 | Yes  Yes | Yes  Yes  Yes | Yes  Yes  Yes | M | 120-150 | 20-40 | Received Praziquantel bath 11/14/18 |
| E12067 | Whitespotted Eagle Ray | Aquarium |  | 3/7/19 | Yes | Yes | Yes | M | 120-150 | 20-40 |  |
| E12066 | Whitespotted Eagle Ray | Aquarium |  | 3/7/19 | Yes | Yes | Yes |  |  |  |  |
| E09017 | Whitespotted Eagle Ray | Aquarium | 2009 | 3/6/19 | Yes | Yes | Yes | M | 120-150 | 20-40 |  |
| E13100 | Whitespotted Eagle Ray | Aquarium |  | 3/7/19 | Yes | Yes | Yes | M | 90-120 | 20-40 |  |
| E12068 | Whitespotted Eagle Ray | Aquarium | 2012 | 3/8/19 | Yes |  |  | M | 120-150 | 20-40 |  |
| E13099 | Whitespotted Eagle Ray | Aquarium | 2013 | 3/6/19 | Yes | Yes |  | M | 120-150 | 20-40 |  |
| E15031 | Whitespotted Eagle Ray | Aquarium | 2015 | 11/11/18  3/5/19 | Yes  Yes | Yes | Yes | F | 120-150 | 40-60 |  |
| E13091 | Whitespotted Eagle Ray | Aquarium | 2013 | 3/7/19 | Yes | Yes | Yes |  |  |  |  |
| E13095 | Whitespotted Eagle Ray | Aquarium | 2013 | 2/28/18  3/5/19 | Yes | Yes  Yes | Yes  Yes | F | 120-150 | 40-60 |  |
| E13094 | Whitespotted Eagle Ray | Aquarium | 2013 | 6/27/18  3/6/19 |  |  | Yes  Yes | F | >150 | 40-60 | Received Praziquantel bath 6/27/18 |
| Cow1 | Cownose Ray | Aquarium | 2009 | 7/3/18 | Yes | Yes | Yes |  |  |  |  |
| Cow2 | Cownose Ray | Aquarium | 2011 | 7/3/18 | Yes | Yes | Yes |  |  |  |  |
| Cow3 | Cownose Ray | Aquarium | 2016 | 7/3/18 | Yes | Yes | Yes |  |  |  |  |
| Cow4 | Cownose Ray | Aquarium | 2016 | 7/3/18 | Yes | Yes | Yes |  |  |  |  |
| Cow5 | Cownose Ray | Aquarium | 2016 | 7/3/18 | Yes | Yes | Yes |  |  |  |  |
| Cow6 | Cownose Ray | Aquarium | 2017 | 7/3/18 | Yes | Yes | Yes |  |  |  |  |
| Cow7 | Cownose Ray | Aquarium | 2018 | 7/3/18 | Yes | Yes | Yes |  |  |  |  |
| Wild water | Wild water | Wild | NA | See Notes | Water |  |  |  |  |  | 5/3/18, 5/4/18 |
| Aquarium water | Aquarium water | Aquarium | NA | See Notes | Water |  |  |  |  |  | 2/26/18, 3/15/18, 3/30/18, 6/21/18, 7/5/18, 11/10/18, 11/29/18, 2/28/19, 3/14/19, |

**Supplementary Table 2.** Number of amplicon sequence variants (ASVs) shared between aquarium and wild whitespotted eagle rays (*Aetobatus narinari*), and aquarium cownose rays (*Rhinoptera bonasus*) for each body site. Shared ASVs are defined as those ASVs detected in at least one wild and one aquarium individual. Proportion refers to the percentage shared out of the total microbial community of each body site. Relative abundance refers to the average relative abundance represented by shared ASVs for each body site. Data were calculated from the ASV table rarefied to 1500 reads per sample.

| **Comparison** | **Body site** | **Number of shared ASVs** | **Proportion of shared ASVs (%)** | **Average relative abundance of shared ASVs (%)** |
| --- | --- | --- | --- | --- |
| Aquarium whitespotted eagle ray  x Wild whitespotted eagle ray | Cloaca | 72 | 13.00 | 0.53 |
|  | Gill | 216 | 15.98 | 0.14 |
|  | Skin | 306 | 17.89 | 0.05 |
| Whitespotted eagle ray x Water | Cloaca | 355 | 39.05 | 0.15 |
|  | Gill | 433 | 24.26 | 0.12 |
|  | Skin | 433 | 20.21 | 0.14 |
| Aquarium whitespotted eagle ray  x Wild whitespotted eagle ray | Cloaca | 72 | 16.90 | 0.64 |
|  | Gill | 216 | 24.74 | 0.12 |
|  | Skin | 306 | 34.77 | 0.20 |
| Aquarium whitespotted eagle ray  x Aquarium cownose ray | Cloaca | 80 | 14.57 | 0.55 |
|  | Gill | 196 | 16.50 | 0.10 |
|  | Skin | 185 | 16.55 | 0.06 |
| Whitespotted eagle ray x Water | Cloaca | 123 | 22.40 | 0.03 |
|  | Gill | 315 | 26.52 | 0.16 |
|  | Skin | 238 | 21.29 | 0.09 |
| Aquarium whitespotted eagle ray  x Aquarium cownose ray | Cloaca | 80 | 10.84 | 0.11 |
|  | Gill | 196 | 19.48 | 0.10 |
|  | Skin | 185 | 23.04 | 0.15 |
| Aquarium cownose ray x Water | Cloaca | 200 | 27.10 | 0.27 |
|  | Gill | 266 | 26.44 | 0.19 |
|  | Skin | 171 | 21.30 | 0.24 |

**Supplementary Table 3.** Number of amplicon sequence variants (ASVs) shared between different body sites for wild and aquarium whitespotted eagle rays (*Aetobatus narinari*), and aquarium cownose rays (*Rhinoptera bonasus*). Shared ASVs are defined as those ASVs detected in at least one wild and one aquarium individual. Proportion refers to the percentage shared out of the total microbial community of each body site. Relative abundance refers to the average relative abundance represented by shared ASVs for each body site. Data were calculated from the ASV table rarefied to 1500 reads per sample.

| **Comparison** | **Body site** | **Number of shared ASVs** | **Proportion of shared ASVs (%)** | **Average relative abundance of shared ASVs (%)** |
| --- | --- | --- | --- | --- |
| Cloaca  x  Gill | Cloaca | 300 | 54.15 | 0.14 |
|  | Gill |  | 22.19 | 0.12 |
| Cloaca  x  Skin | Cloaca | 350 | 63.18 | 0.13 |
|  | Skin |  | 20.47 | 0.05 |
| Gill  x  Skin | Gill | 662 | 48.96 | 0.06 |
|  | Skin |  | 38.71 | 0.04 |
| Cloaca  x  Gill  x  Skin | Cloaca | 237 | 42.78 | 0.18 |
|  | Gill |  | 17.53 | 0.15 |
|  | Skin |  | 13.86 | 0.07 |
| Cloaca  x  Gill | Cloaca | 217 | 50.94 | 0.23 |
|  | Gill |  | 24.86 | 0.15 |
| Cloaca  x  Skin | Cloaca | 252 | 59.15 | 0.21 |
|  | Skin |  | 28.64 | 0.21 |
| Gill  x  Skin | Gill | 376 | 43.07 | 0.09 |
|  | Skin |  | 42.73 | 0.18 |
| Cloaca  x  Gill  x  Skin | Cloaca | 165 | 38.73 | 0.30 |
|  | Gill |  | 18.90 | 0.16 |
|  | Skin |  | 18.75 | 0.31 |
| Cloaca  x  Gill | Cloaca | 275 | 51.115 | 0.136 |
|  | Gill |  | 37.162 | 0.118 |
| Cloaca  x  Skin | Cloaca | 245 | 45.539 | 0.146 |
|  | Skin |  | 38.766 | 0.143 |
| Gill  x  Skin | Gill | 287 | 38.784 | 0.115 |
|  | Skin |  | 45.411 | 0.133 |
| Cloaca  x  Gill  x  Skin | Cloaca | 199 | 21.215 | 0.173 |
|  | Gill |  | 15.645 | 0.145 |
|  | Skin |  | 20.431 | 0.163 |

**Supplementary Figures**

**
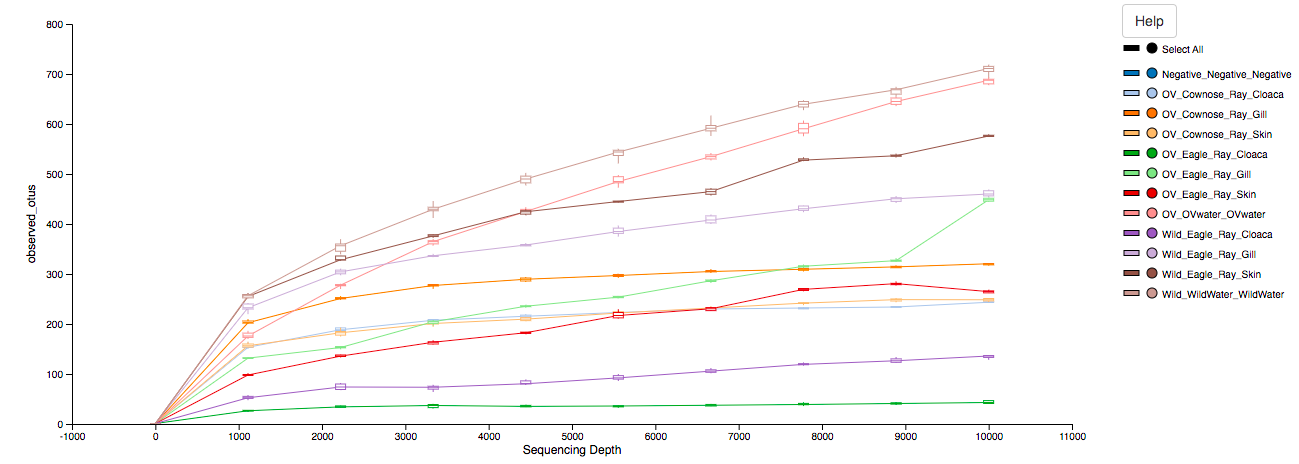

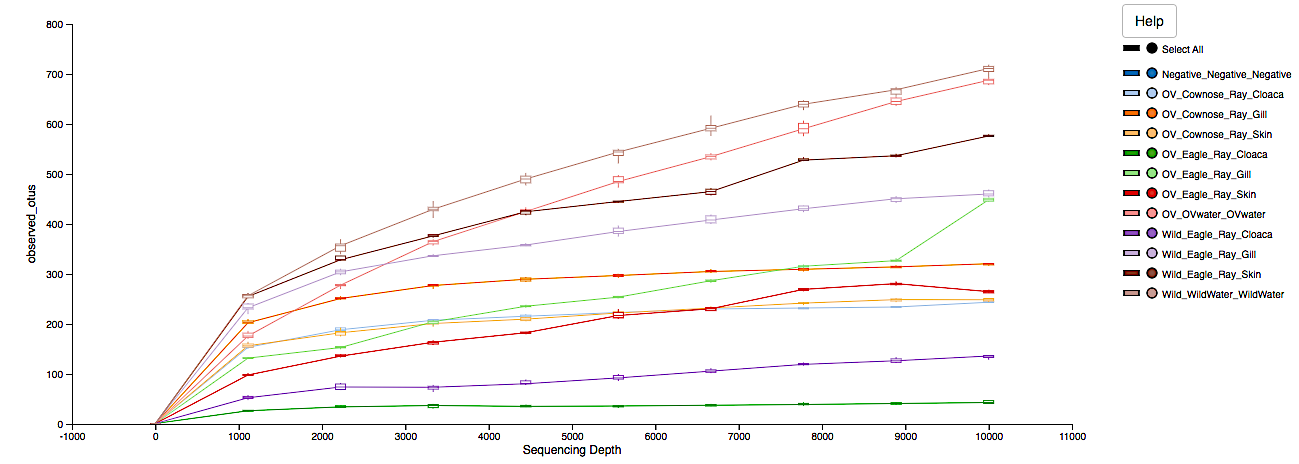
**

Observed ASVs

Sample Type

Aquarium cownose ray cloaca

Aquarium cownose ray gill

Aquarium cownose ray skin

Aquarium spotted eagle ray cloaca

Aquarium spotted eagle ray gill

Aquarium spotted eagle ray skin

Aquarium water

Wild spotted eagle ray cloaca

Wild spotted eagle ray gill

Wild spotted eagle ray skin

Wild water

0

**Supplementary Figure 1.** Rarefaction curves for the cloaca, gill, and skin samples for wild and aquarium whitespotted eagle rays (*Aetobatus narinari*) and aquarium cownose rays (*Rhinoptera bonasus*). Curves are based upon the number of observed amplicon sequence variants.

**Supplementary Figure 2.** Principal coordinate analysis (PCoA) based upon a Bray-Curtis dissimilarity matrix between a) cloaca, b) gill, and c) skin samples for wild and aquarium whitespotted eagle rays (*Aetobatus narinari*) and aquarium cownose rays (*Rhinoptera bonasus*). Wild whitespotted eagle rays harbor different microbial communities than aquarium whitespotted eagle rays, as indicated by the separate clustering. Microbial communities from cownose rays appear more similar to aquarium whitespotted eagle rays than those from the wild.

**Supplementary Figure 3.** Bar plots representing the relative abundance of the top 20 most abundant microbes associated with a) the cloaca, b) the gills, and c) the skin of aquarium cownose rays (Rhinoptera bonasus), aquarium whitespotted eagle rays (Aetobatus narinari), and wild whitespotted eagle rays. Skin and gill communities appear distinct between aquarium and wild whitespotted eagle rays, while cloaca samples are more similar. “NA” indicates that classification to the species level was not possible.


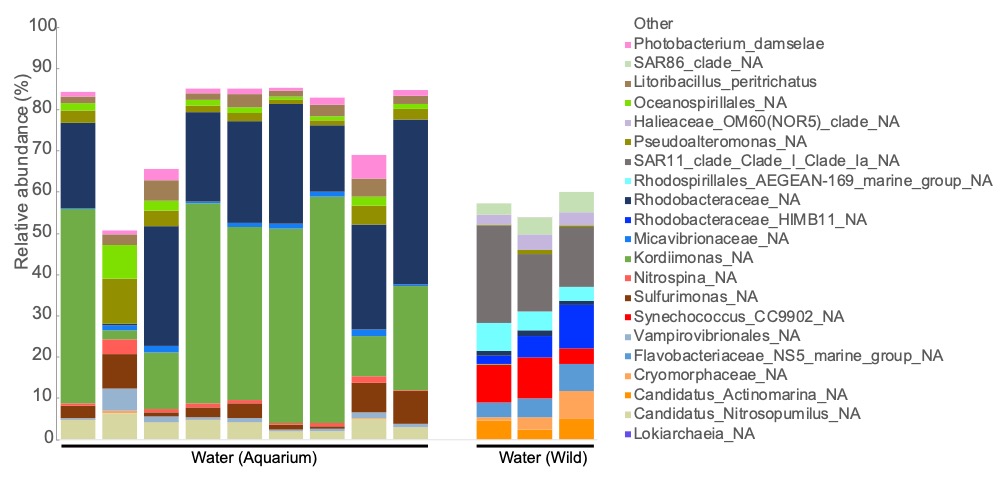


**Supplementary Figure 4.** Bar plots representing the relative abundance of the top 20 most abundant microbes associated with the surrounding water column of the wild (Sarasota Bay, FL) and aquarium (Ocean Voyager of Georgia Aquarium) environments. “NA” indicates that classification to the species level was not possible.

**Supplementary Figure 5.** Alpha diversity metrics of a) observed amplicon sequence variants (ASVs), and b) Shannon diversity indices, for all body sites (cloaca, gill, and skin) of aquarium cownose rays (*Rhinoptera bonasus*)*,* aquarium and wild whitespotted eagle rays (*Aetobatus narinari*), as well as the surrounding water column. The cloaca microbiome for all three ray categories had lower diversity compared to other body sites.

**Supplementary Figure 6.** Principal coordinate analysis (PCoA) based upon a Bray-Curtis dissimilarity matrix between cloaca, gill, skin, and water samples for aquarium cownose rays (*Rhinoptera bonasus*). Microbial communities associated with all body sites cluster separately from the surrounding water, but do not differentiate according to body site.
